# Marginalising prevention during Health Devolution in Greater Manchester? A qualitative study of the perceptions and experiences of public health professionals

**DOI:** 10.1093/pubmed/fdag050

**Published:** 2026-07-03

**Authors:** Chris White, Daniel Bloyce, Miranda Thurston, Paul Bissell

**Affiliations:** Wrexham University, Mold Rd, Wrexham, LL11 2AW, Wales; University of Chester, Parkgate Rd, Chester, CH1 4BJ, England; University of Inland Norway, Terningen Arena, Fakultet for helse- og sosialvitenskap, Hamarvegen 112, 2406 Elverum, Norway; University of Chester, Parkgate Rd, Chester, CH1 4BJ, England

**Keywords:** devolution, devolved health, population-based and preventative services, management and policy, public health

## Abstract

**Background:**

There are increasing moves across the globe towards devolution of various state services, including health. However, Greater Manchester (GM) is the only region in England with fully devolved health powers. A key feature of the policies supporting devolution in GM was a proposed uplift in focus and funding for prevention of ill-health. This was deemed important for supporting people’s health and wellbeing, and economic stability. This study explores the experiences of people working in public health during devolution in GM, and seeks to understand the constraints and opportunities of this novel approach.

**Methods:**

Forty two semi-structured interviews were conducted with people in public health roles in GM, examining how relational processes contributed to how prevention was perceived and enacted.

**Results:**

Prevention work was considered less of a priority than treatment-centred activities in GM, both in terms of planning and practice. Some plans for increased prevention were neglected, and lacking direction, which tended to lead to stagnation of genuinely innovative prevention-based plans.

**Conclusions:**

Continued marginalization of prevention could be explained through processes such as lifestyle drift and the enduring prestige attached to treatment work. Despite the intentions behind the policies, there were instances where devolution seemed to exacerbate these tendencies.

## Introduction: shifting (back) towards prevention?

Throughout modern history, public leaders have, to a greater or lesser extent, acknowledged that health outcomes are strongly related to factors beyond health services.^1–3^ Such factors range from economic policy decisions^4^ to the design of people’s neighbourhoods.^5^ Referred to collectively as the wider, or social, determinants of health, the term conceptualizes the ways in which environments can be transformed to create the conditions for health.^6^ This often requires intersectoral action if the conditions for health are to be created.^7–9^ However, researchers have argued that whilst UK health policies have contained many shifts in rhetoric towards the social determinants and health promotion, the dominant funding models have been set-up to prioritize primary and secondary care, and a biomedical approach to health focused on treatment over prevention.^10^ Some argue that this process has not just taken place within the publicly funded National Health Service (NHS), but also, to varying degrees, in public health.^11^^,^^12^ Whilst public health researchers have focussed on the social determinants of health, there has been an increasing trend for governments to address these issues through (often short-term) interventions, and the introduction of observable key performance targets for public health teams, such as setting targets for contacts made with a particular group of people.^13–15^ However, critics suggest that some ‘target hitting’ approaches may mean the focus becomes more firmly placed on hitting the target, rather than the impact of work on potential outcomes.^16^^,^^17^ Many have linked these trends to processes of medicalisation,^18–21^ which has tended to displace prevention to the periphery of health policy and practice, keeping the focus on targets and treatment.^15^ This also deflects attention away from a concern with prevention and narrowing social inequalities in health.

Others have also argued that concomitant with medicalization, there has been a trend towards individualism, which emphasizes personal responsibility for individual lifestyle choices and health outcomes.^22^^,^^23^ The focus on individualism in the UK has been linked to the dominance of a neo-liberal ideological framework and related policies and strategies since Margaret Thatcher’s time as Prime Minister.^24^ Neoliberalism has endured, to a greater or lesser degree, in British politics ever since.^25^ As an ideology, it is tied to the value of freedom of choice for people, marketization and financialization.^26^ It sees the welfare state and trade unions as unnecessary encumbrances and encourages the idea of a neoliberal subject, responsible for their position in the global marketplace.

Critics of neo-liberalism also argue that in policy and practice, the focus on individual responsibility contributes to obscuring the role of structural approaches concerned with the wider determinants of health and health inequality.^27^ A corollary of individualism is that individuals are ‘blamed’ for their poor health, shaming the ‘failing’ majority.^28^ Neo-liberal approaches can, therefore, be at odds with work on the wider determinants of health, since they tend to emphasize reduced state spending and transfer of services to private sector providers. Yet, greater national and local government intervention is required if environments and conditions are to be shaped to support health.^29^^,^^30^ Such debates are common in public health, where the concept of ‘lifestyle drift’^31^^,^^32^ is used to refer to the tendency for policies and strategies to start off by paying lip service to ‘the need for action on upstream social determinants of health inequalities only to drift downstream to focus largely on individual lifestyle factors’^31^ (p. 148). However, there is limited research on the implementation of prevention-focused policies during a period of major organizational devolution. This study addresses this gap by focusing on the perceptions and experiences of public health professionals during the Greater Manchester (GM) health devolution deal. This deal was presented as a new and ‘radical’ way of improving health, with prevention—working on the wider determinants—being positioned as a ‘central pillar’ of policies.^33^

Amidst an era of growing rhetorical emphasis on intersectoral collaboration and action in the UK, there has been a growing trend towards devolution, referring to the transfer of legislative and financial powers away from the nation-state towards a chosen body, or bodies, at a more local or regional level. This trend is likely to increase given that the current Labour government in the UK has publicly promised even more devolution deals.^34^ Previous research has revealed both opportunities and challenges from such changes. In terms of opportunities for prevention work, there may be greater opportunities for collaboration and, ultimately, greater joined-up thinking on the wider determinants of health, with key decision-makers being pulled closer together. As for challenges, there are concerns that the national government may not allocate adequate funding to focus on prevention locally and that devolution itself may add further layers of political complexity that limit change and collaboration.^35^ To the best of our knowledge, the current study is the first to explore ill-health prevention specifically within the context of devolution. However, it builds on work by Walshe *et al*.^36^ who suggested that the health and social care reforms in GM might best be described as ‘“soft devolution” because, unlike most devolution reforms, they have no statutory basis. They are, essentially, an agreement for administrative delegation between the Department of Health and Social Care, national bodies like NHS England and NHS Improvement, and NHS organisations and local authorities and others in Greater Manchester’ (p. 6). For Walshe *et al*.^36^ this meant that policy priorities for devolution resembled NHS mandates for other areas, and the key difference was therefore not so much about autonomy over policy, but implementation, with a greater focus on collaboration in GM than other health networks in the UK. The extent to which this can be realised is important, as the above literature suggests that, historically, much ‘lip service’ has been paid to an increased focus on prevention through increased collaboration on the wider determinants, but in the UK this has rarely been met through implementation. By focusing on the experiences of key stakeholders in GM, this paper seeks to uncover how discourses of prevention were manifested during devolution in GM.

### Health devolution in GM

Prior to health devolution, the 2012 Health and Social Care Act contributed to what many considered to be the largest reorganization of public health in England since the NHS was formed in 1948.^37^ Most prominently, Primary Care Trusts (PCTs) were reworked into two separate strands.^38^ Previously, PCTs were in charge of the majority of public health services, as well as commissioning (strategic planning and purchasing) most other health services at a local level.^39^ However, under the 2012 Act public health services and public health staff [led by local Directors of Public Health (DsPH)] were largely transferred to local authorities, whilst the commissioning of health services was transferred to newly created local (General Practioner-led) Clinical Commissioning Groups (CCGs). This was the first time that public health services and teams had been in local authorities in the UK since 1974. This reorganization was very much branded as public health returning ‘home’, with many seeing public health most appropriately placed in local authorities, where they were better able to influence strategies in areas beyond health services in relation to the wider determinants of health.^40^^,^^41^ Public health groups in GM were still managing these changes at the onset of the devolution deal.

Health devolution in GM (which first came into force mid-way through 2017) was completely unique in England, as it was, and remains, the only complete devolution of health and social care strategy and funding to a region. This deal involved taking charge of a £6 billion annual health and social care budget from national government. This budget was based on the pooling of what was previously spent on health and social care by NHS clinical commissioners and local councils in GM. On top of this a £450 million ‘transformation fund’ was created to assist with implementing changes over the first five years of health devolution.^42^ As part of the deal, 37 different organizations (including all local authorities, NHS Trusts, and Clinical Commissioning Groups in GM) signed a memorandum of understanding for shared working priorities. The Greater Manchester Health and Social Care Partnership (GMH&SCP) was also created to coordinate policy and decisions on health and social care in GM. The 37 different organizations were represented in this group, and a Chair and Executives were identified, all of whom had been working in health and social care in GM previously. From the outset, devolution policy documents outlined a predicted £2 billion shortfall in health care funds if no changes were made. It was therefore suggested that ‘radical change’ was needed via devolution, especially with regard to the balance between prevention and the high-cost treatment services (This is particularly important since the £450 million Transformation Fund equated to less than 25% of this predicted shortfall)^33^ (p. 6). It appeared that a segment of the transformation fund would be used to support this. Other areas prioritized were community-based care and support, standardizing acute and specialized care and support, and back-office services.^33^

At the onset of devolution GM had a population of around 2.8 million, making it the second most populous urban area after Greater London in the UK.^43^ In terms of health outcomes in GM, Public Health England data ‘put Greater Manchester at or near the bottom for a whole range of indicators’^44^ (p. 352). This was recognized in early health devolution policies, where it was stated that ‘the health outcomes for GM people are worse than those in other parts of the country and health inequalities are deep-rooted’^33^ (p. 6). These outcomes were linked to income inequalities in GM, as the region had higher than average levels of overall deprivation and some of the most deprived areas in the UK,^45^ with ‘36% of Greater Manchester population’ living ‘in 20% of the UK’s most deprived neighbourhoods’^46^ (p. 4).

## Methods

This study was part of a PhD on devolution and prevention in GM.^47^ The research question for this specific study was: How does prevention feature in the aims and purposes of GM public health networks? A qualitative approach was used, which consisted of 42 semi-structured interviews (25–75 minutes) with people in various roles related to public health and prevention. Participants were selected via purposive sampling,^48^ designed to select professionals and practitioners from across public health networks in GM. The sampling process was initially guided by documents from the National Institute for Health and Care Excellence, which provided guidance on those in local public health roles who should contribute to environmental-based prevention work. People with these roles in five different boroughs of GM were contacted. From this initial sample, snowball sampling was used to identify others deemed to have a role in the development and implementation of public health in GM (see Table 1). The sampling strategies were deliberately non-random to ensure that participants had personal experiences of topics related to the aims of the research. Interviews were conducted during the first two full years of implementing health devolution (2017–2019), and focused on the following topics: learning about the participant and their professional role; views and experiences of prevention in GM; activities for prevention in GM; organizational priorities; organizational changes and transitions; working with others in GM. Interviews were conducted until no new issues or concepts emerged. With consent, the interviews were audio-recorded and transcribed verbatim. Ethical approval was granted by the University of Chester Faculty Ethics Committee.

**Table 1:** Research sample.

Role (Pseudonym)	Regional (GM) role?	Leadership role?	Sex	Age	Self-assessed physical activity level	Physically active during work commute?
(# = Borough; S = Snowball; A = Additional borough)	(31% = Y)	(57% = Y)				(26% = Y; 26% = Sometimes)
Public Health Consultant (1)	N	N	Male	50–59	Moderate	Y
CCG Lead (1)	N	Y	Male	50–59	High	Y
Public Health Consultant—Age Friendly (1)	N	N	Male	20–39	High	Y
Councillor—Cycling Champion (1—S)	N	N	Male	50–59	High	Y
Councillor—Active Travel (1—S)	N	Y	Female	50–59	High	Y
Transport Policy Officer (1—S)	N	N	Male	20–39	High	Y
Councillor—Health & Wellbeing (2)	N	N	Female	60–69	Moderate	N
Public Health Consultant (2)	N	N	Male	50–59	High	Sometimes
Chief Executive; Health Lead (2)	Y	Y	Male	40–49	Moderate	Y
Public Health Program Officer (2)	N	N	Male	20–39	Moderate	N
Councillor—Children & Families (2)	N	N	Male	60–69	High	N
Local Sport Org.—Chair (2—S)	N	Y	Male	40–49	Moderate	N
Local Sport Org.—Development Officer (2—S)	N	N	Male	40–49	Moderate	Sometimes
DPH (3)	N	Y	Female	50–59	Moderate	Sometimes
Director of Care and Integration (3)	Y	Y	Male	40–49	Moderate	N
Councillor—Health & Wellbeing (3)	N	Y	Male	60–69	Light	N
Councillor—Children & Families (3)	Y	Y	Female	40–49	Moderate	N
Head of Public Health & Leisure (4)	N	Y	Male	40–49	Moderate	N
Councillor—Public Health (4)	N	Y	Male	60–69	Light	N
Health Advisory Group—Chair (4)	N	Y	Male	40–49	Moderate	Sometimes
Councillor—Health & Wellbeing (5)	N	Y	Male	60–69	Light	N
PA Consultant (5)	N	N	Male	40–49	High	Sometimes
Councillor—Adult Social Care (5)	N	N	Male	60–69	Moderate	N
DPH (5)	N	Y	Male	60–69	Moderate	Y
CCG Lead (5)	N	Y	Female	50–59	Moderate	Sometimes
Councillor—TFGM Member (5—S)	Y	N	Male	60–69	High	Sometimes
Senior Public Health Nurse (5—S)	N	Y	Female	50–59	High	N
Local Sports Org.—PA & Health Chair (5—S)	N	Y	Female	20–39	High	Sometimes
Health & Social Care Development Lead (GM)	Y	Y	Male	40–49	Moderate	N
Public Health Advisory Group—Regional Director (GM)	Y	Y	Female	50–59	Moderate	Sometimes
Public Health Advisory Group—Regional Lead (GM)	Y	Y	Female	40–49	High	Sometimes
Public Health Advisory Group—Consultant (GM—S)	Y	N	Male	20–39	Moderate	N
Regional Sport Org.—Development Officer (GM—S)	Y	N	Female	40–49	Moderate	N
Regional Transport Org.—Head of Logistics (GM—S)	Y	Y	Female	20–39	High	Y
Regional Transport Org.—AT Officer (GM—S)	Y	N	Female	40–49	High	N
National Cycling Org.—Partnership Manager (GM—S)	Y	Y	Male	40–49	Moderate	N
Town Planning Officer (A1—S)	N	N	Male	20–39	Moderate	N
Public Health Consultant (A1—S)	Y	N	Female	20–39	High	N
Sport and PA Officer (A2—S)	N	N	Female	20–39	Light	N
DPH (A2—S)	N	Y	Female	50–59	Moderate	Y
CCG Lead (A2—S)	N	Y	Male	50–59	High	Sometimes
Local Sport Org.—Chair (A2—S)	N	Y	Female	40–49	Moderate	Y

Reflexive thematic analysis (RTA) was used to analyse the data.^49^^,^^50^ Analysis began by reading through transcripts for data familiarization. This was then followed by a process of coding, which involved identifying, highlighting and refining codes within, and across, the transcripts. These codes were developed to capture different ideas identified in the data set. Refining the codes was a process that sought to prioritize building a ‘story’ from the data, rather than trying to ‘fit’ the results into ideological preferences.^49^^,^^50^ As analysis continued, this story was discussed with PhD supervisors, who were more detached from the immediacy of the process and able to guide debates on how the data could be theorized. The analytic process was supported by the use of NVIVO software, which enabled efficient management of participants’ quotations, and assigned codes. Formalized codes were then related to each other, and broader analytic categories (or sub-themes) were developed. The next stage involved combining categories into overarching analytic themes that would shed light on the research question. These ‘candidate themes’ were reviewed and refined to check that there were sufficient relevant data to support them and that they appropriately represented the data set as a whole.^50^

According to Braun and Clarke^49^ (p. 79) RTA ‘minimally organises and describes your data in (rich) detail. However, frequently it goes further than this, and interprets various aspects of the research topic’. It is the latter that leads to the development of what Braun and Clarke^50^ (p. 58) label fully realized themes, which are described as the overall purpose of RTA: to identify ‘patterns of shared meaning underpinned by a central organising concept’. In other words, the themes that were developed here provide a ‘story’ of the meanings behind the data. The two themes most relevant to the research question for this paper were: (i) medical prestige, alongside marginalizing and stigmatizing public health; and (ii) the double-bind of finding direction for prevention in GM. The findings are reported in accordance with Levitt *et al*’.s ^51^ Reporting Standards for Qualitative Research, which outlines major components that should be reported in a qualitative research paper.

## Results

### Medical prestige, alongside marginalizing and stigmatizing public health

When participants were asked how they saw health devolution facilitating a greater focus on prevention, they typically described the historical approach to public health in GM as influential. The rhetorical shift towards prevention was often labelled radical in health devolution policies, and participants saw this as relating to previous approaches to improving health in GM, in that prevention had long been viewed as a ‘fringe’ issue amongst most health organizations. This was to the extent that, historically, a very small share of health funding had been attributed to public health in GM, and the vast majority of the activities delivered were also treatment-based (treating issues such as substance misuse and sexual health). However, despite the prevention-based focus of devolution policies, it was speculated that these processes were likely to continue, as few expected the focus on treatment approaches (as well as the high share of funding) to suddenly subside. This meant that much of the new funding, such as the Transformation Fund, was directed away from what was considered ‘true’ prevention—that being work which focused on the wider determinants. Data that led to these findings are shared in Data Box 1.

**Box 1**

Many participants argued that the core agenda of GM health leaders had previously been dominated by hospital care, with a focus on making such services more efficient: ‘When you talk about the health service, people always talk about hospitals, they don’t talk about primary care or anything else’ (Public Health Consultant 1). Participants argued that this clinical focus had substantially limited the work of public health teams, with many of their proposals and requirements being ‘overshadowed’ by clinically-focused activities of the NHS:

‘I guess we get distracted by hospital services and the wrong end of the healthcare spectrum really. It is inevitable that you have to spend a lot on interventions through hospital services. But, usually, there is nothing left over. It has all been spent and little is left over’ (CCG Lead 1).

Participants recognized the necessity of substantial expenditure on hospital care but were ‘dissatisfied with the ways in which funding had previously been distributed’. Those from public health groups suggested that leaders had developed a hierarchical model whereby the needs of hospitals were addressed first, leaving public health teams to manage any remaining funding. There were even instances, historically, in which public health funds were cut to support hospital work. For example, some participants noted that when public health was positioned within the NHS-led PCTs there had been a series of cuts that were designed to help hospital budgets:

‘The year before I inherited the public health system, the PCT at the time cut the public health budget by a third to invest in hospital beds. A third! So that was just about propping up a hospital by stripping out intervention-prevention’ (Chief Executive; Health Lead 2).

This was prior to the 2012 Health and Social Care Act, a policy which led to responsibility and budgets for public health transferring to local authorities in England. Participants were critical of how public health was dealt with under the previous system, as they felt that it had been neglected and underfunded:

‘You realise how shockingly bad it [public health] has fared in the NHS since 1974. So, the budgets are small, that is the first thing. Because the NHS, it treats people when they are ill, so public health must not have been a priority for them [the government]. So only one pound in twenty was spent on public health in the NHS. It was a Cinderella service for them’ (Chief Executive; Health Lead 2 ).

Those previously employed by the PCTs in GM initially felt positive when hearing that public health would be moving into local authorities under the 2012 Act. However, these changes coincided with the Coalition government’s cuts to public services, where local government was among the worst affected.^52^ This resulted in DsPH having fewer resources, in real terms, when housed within local authorities than under the previous system of PCTs: ‘You are arriving and everyone is pleased to see you and saying we can do more, and then actually you have to do that with less’ (Public Health Consultant 2). Local authority public health officers were therefore having to manage expectations about the extent to which they would be able to make changes to their working practices, and there were fears this would continue under devolution.

A large proportion of the funding that public health teams had received prior to devolution had been spent on what many viewed as treatment activities:

‘About 90% of spend in public health is actually treatment-based, so it is spent on alcohol, drugs, etc. So, it is not spent on public health as I have perceived it to be. It is spent on dealing with people who need treatment, which is what the NHS does. [They have] stripped the money out of public health, and what is actually left in public health has pretty much been medicalised’ (Public Health Consultant 2).

This meant that most public health work from local authorities closely resembled that of clinical NHS bodies, with all health organizations focusing on treating illnesses via specific services. Participants did not dispute the importance of such services in public health; rather, the point of contention was that public health leaders were not being given enough resources to work beyond them, thus limiting their capacity to focus on prevention. This meant that the main services provided by public health teams were distanced from what some participants described as ‘true’ public health:

‘The funny thing is, a lot of those aren’t preventive services. Drug and alcohol are treatments, sexual health is largely testing, and secondary prevention, so, once people have had an infection. There is a preventive bit with community organisations, but a lot of it is not what we would call pure public health or prevention’ (Public Health Consultant 1).

Participants considered that ‘true’ or ‘pure’ public health was aimed at preventing poor health by addressing the wider determinants of population health. Those who discussed public health in this way suggested that it was most appropriate that public health teams were enabled to assist organizations in areas such as housing, education, and transport, to identify and work towards health outcomes in their various strategies. However, this was largely constrained in local authorities.

For public health officers, the perceived bias towards clinical services went beyond funding decisions, even once devolution was in place. For example, some also cited occasions when they felt not only marginalized, but also stigmatized by other individuals from wider health networks: ‘When I first took on this role, people were saying to me “public health, where did it all go wrong?”’ (Regional Health & Social Care Development—Director [GM]). It was perceived that such stigmatization, and the wider processes that contributed to it, were influencing regular communication channels for devolution plans. For example, in development meetings and forums public health officers felt their comments were not afforded the same attention as those from treatment-centred health networks: ‘The first question I am asked, “are you a clinician”? And already, [because I am not] half the room will switch-off to you’ (Public Health Advisory Group—Regional Lead [GM]). Participants felt this was because public health activities were not afforded the same prestige as the services delivered by clinically-focused providers, with those in wider health networks often dismissing or trivializing work produced by someone from a non-clinical background. This was considered rather problematic when holding meetings on how the make-up of GM health provision following devolution should look with various new partners. Indeed, there were suggestions that perceptions of clinical work, as well as greater familiarity amongst clinical groups, had meant that tasks such as hospital reforms under devolution were developing at a much faster rate than work on prevention.

When trying to explain these processes, some participants felt that there was a perception amongst many clinical workers that public health teams have, historically, not been able to demonstrate significant improvements in health outcomes clearly:

‘There is probably a responsibility that lies with those who are working within public health. They weren’t being seen to be very effective in what they were doing. There was a lot of observing and measuring, but little action on public health to improve those outcomes… or influence to change, to affect, those who can make the changes to public health’ (CCG Lead 1).

This suggests that many people in wider health networks in GM continued to prioritize work that had more immediate and, therefore, more visible influences in the short-term. However, some noted that this was more difficult for public health teams, with most of their activities designed to facilitate long-term outcomes, many of which were difficult to measure. In other words, it was perhaps easier to trace the quantitative measures of direct care than the influences of services which prevent people from requiring greater assistance from the healthcare system in the first place:

‘For instance, mental health problems should drop very rapidly with exercise. So, contacts at the GP [General practitioner surgeries] will hopefully fall. But then it will get filled with something else. So, it is very difficult to prove. It is the right thing to do, we all know it is the right thing to do, but it is very difficult to prove it is the right thing to do when you are looking at data in terms of your outcomes’ (CCG Lead [A2—S]).

This illustrates that prevention work was often considered according to medical models of evaluation, with less reflection on the extent to which this could be a more appropriate means of evaluation going forward.

These findings illustrate the prestige attached to medical ways of working, which appeared to remain prominent through devolution. Therefore, much work was required to reframe public health and prevention; however, participants rarely discussed examples of how such re-framing could take shape.

### The double-bind of finding direction for prevention in GM

Participants regularly argued that both health devolution and the policy shift towards prevention had come at the ‘wrong time’ from a financial perspective, especially when it was felt expansion opportunities were much greater prior to 2010 when the government’s national austerity measures were brought in. However, participants stated that it was only through devolution that they had started to see a more serious political shift towards prevention in GM. The difficulty was that whilst it was suggested that there was ‘new’ funding for health in GM under transformation-based funds, the overall budget actually meant that there was less to spend for many groups.

Without any great sense of accountability (for prevention) under the devolved arrangements there was a feeling that the development of ‘true’ prevention work in GM would continue to be sporadic and incremental, despite the strong rhetoric associated with devolution. Data that led to these findings are shared in Data Box 2.

**Box 2**

It was hoped that the transformation itself would make up for reductions: ‘I suppose what we would really like is to have more money to spend on the prevention element of stuff … the lifestyle, trying to work on health inequalities. There is no foreseeable option of that happening because the policy is about cutting it all back’ (Public Health Consultant 1). Leaders in GM were therefore placed in something of a double-bind, in that they recognized that prevention work would likely be one of the best ways of reducing their overall spend, at least in the long-term, yet, even with devolution policy and opportunities for new funding frameworks, the pressures on hospitals and social care meant that they saw few opportunities to substantially alter the available funding allocations towards prevention:

‘I think it is harder and harder for public services to invest in prevention. At a time when, for example, the social care system is in such a hugely financially challenged time, it is hard to make the case. Ironically [though] it is the prevention agenda that is actually going to make the difference’ (Director of Care and Integration 3).

This meant that there was little change to the overall funding picture. Added to this complexity was the perceived prestige attached to clinical work. In this context, some health leaders in GM showed a more sympathetic view of the outputs of public health teams, as they felt that influences on the wider determinants of health could come from other, external, public service departments, such as housing:

‘If we were talking about what does investment in public health look like, you would look at the state of our housing stock in GM and what is the strategy to improve that. That is public health, but it is not in anyone’s budget lines as public health’ [Regional Health & Social Care Development—Director (GM)].

For some GM health leaders, public health teams only constituted a segment of public health work in the region, with wider health determinants work falling under the remit of other departments and organizations. However, there was little indication from participants of how health devolution may alter, or influence, such issues in practice. The same might be said for devolution policy documents, which all contained a push towards prevention and the wider determinants, but with few details, especially when compared to policy messages for treatment work. This lack of direction was challenging for those working on prevention in GM, especially when trying to find room amongst a contested funding/policy space, with lots of organizations wanting a say on how health should look under new devolved arrangements.

Importantly, many suggested that there were few opportunities to use the devolution Transformation Fund and other associated budgets on more ‘innovative’ prevention approaches. To exemplify this, participants from local authorities felt that the bids they had submitted to the Transformation Fund panels were quite ambiguous. This was in response to an understanding that they were only being asked to provide summaries of intended work:

‘You wouldn’t really know what was envisaged [just] from seeing the bid, certainly not in any sort of detail. But that is what they want at this point, the sort of overview of the bid. The person in our team who has been working on it says it is only a real summary’ (Public Health Consultant 1).

With bidding (and funding dissemination) approached in this manner, participants expressed concern that those who received funding from the GMH&SCP had little to be held accountable for and may, therefore, be able to use the money to work in similar ways as before (where there were already pressing needs for treatment). This perceived ambiguity of bids was particularly important since participants highlighted that the statutory services for local authorities, which remained from the national Health and Social Care Act seven years prior, were only remotely related to prevention. Some feared that, without further direction from the GMH&SCP, the Transformation Funds would do little to advance this. Those most ‘convinced’ by the evidence supporting prevention therefore argued that leaders from both GM and national government would need to consider greater enforcement on local authorities if they genuinely wanted public health teams to consistently develop programs that challenged these deeply embedded ways of thinking: ‘People talk about prevention all the time, and the government talks about prevention all the time and then doesn’t necessarily follow through. A key part of prevention is often primary legislation and that needs to come from government level’ [Public Health Advisory Group—Regional Director (GM)].

## Discussion

### Main findings of this study

Discussions about acting on the wider determinants of health were largely absent in interviews, with participants feeling constrained to prioritize treatment approaches within GM. This suggests that prevention work may be much harder when there are very significant social inequalities, as leaders found it difficult to look beyond immediate health needs. This is just one of several double-binds in these findings, where long-term assumptions and practices, as well as the obvious demands on services, meant that health leaders and politicians continued, in the main, to turn their focus away from prevention, but in doing so were potentially exacerbating demands. Prevention-based developments in GM were further constrained by the enduring prevalence of treatment-oriented perspectives. Drawing on Zola,^18^ we interpret this constraint as reflecting long-term processes of medicalization. In this study, it was apparent that medical-based services of the NHS were viewed favourably by people within wider health networks to the extent that medicalized practices had become the norm in areas of health work beyond the NHS. Coinciding with this, prevention work was perceived to manifest itself in terms of lifestyle drift. Despite the intentions of devolution, it was speculated that this process was likely to continue, as few expected the focus on treatment to suddenly subside, which meant that much of the new funding, such as the Transformation Fund, would be directed away from ‘true’ prevention.

### What is already known on this topic

The Chief Officer for the GMH&SCP delivered a public lecture in 2020, where it was noted that, despite data suggesting an increase in deprivation within GM during the first period of devolution, there were improvements in areas such as the number of people smoking, the number of unsafe births, the region’s mental health offer, cancer survival rates, social prescribing, and the number of people living ‘independently’.^53^ It, therefore, might be concluded that GM’s prevention offer was improving. However, the extent to which this reflects a commitment to working on the wider determinants—so-called ‘true’ public health—is debatable. Indeed, despite the apparent opportunities afforded by devolution, our findings suggest that this rhetoric did not change priorities in GM, in that resources continued to be disproportionately allocated to treatment services. These are key issues that others, such as Walshe *et al*.^36^ have identified in GM, as they observed ‘no major shifts in expenditure away from hospital care to support the population health agenda’ (p. 43). Adding to this complexity, plans for prevention were lacking in specific detail when compared to treatment work in GM. This is consistent with some other studies, which have demonstrated that there are sometimes difficulties in defining prevention-based concepts when working with a range of partners, many of whom may have limited experience of prevention based on the wider determinants of health, and how it may look in practice.^54^^,^^55^

### What this study adds

This paper has identified important interweaving processes when explaining the enduring focus on treatment and secondary prevention in GM. As previous research suggests, the more established nature of clinical work in GM was greatly influential, however, we have also found that this was exacerbated by both the non-specific nature of prevention, and the complexity of large-scale organizational change.

We firstly covered how people in more clinical roles did not always consider the skills of those working in wider public health networks to carry the same weight as those with clinical expertise. This is also consistent with past research that has found that those with a clinically informed or specialized background often have greater credibility within partnerships.^56^^,^^57^ We have labelled this ‘medical prestige’, a concept referring to the ways in which medical-based practices were often viewed as having more status. Even under a significant opportunity for change, through the development of many new partnerships under devolved responsibility for health services, it appeared to be difficult to alter power dynamics that had historically favoured clinical services. As an example of this, it was clear that the more established group in this context (those in clinical roles) had greater degrees of influence over standards and direction. This became problematic, as prevention was being pushed as something radical and somewhat different, yet it appeared that wider determinants work was still being largely viewed under medicalized frames of reference (with a focus on accountability and short-term evaluation). This may have meant that prevention work was being ‘set up to fail’. Indeed, it might be argued that prevention work was subject to a double-bind in relation to measurement of impact, in that there was little overt critique, amongst those in clinical roles, on the extent to which evaluation models were appropriate for prevention; yet a perceived lack of progress on standards, largely set by others, was being used as the reason for the marginalization of those working on prevention, and thus their limited influence on standards and direction.

Despite efforts to change the situation in GM, there was continuity of previous pressures and practices seen under national and NHS ‘rule’, which constrained attempts to draw partnerships together in the name of prevention. This suggests that major organizational change, such as devolution, intended to bring about partnerships, can actually give rise to processes that (continue to) push some people and issues to the periphery. Indeed, it might be argued that those working on the wider determinants of health also experienced greater ‘messiness’ than those in clinical roles, many of whom were part of clearer networks and more established ways of working. However, those working on prevention were negotiating many new partnerships, whilst also working with fewer details of how they might work across the new health region. Furthermore, those in public health roles were still addressing the changes of the 2012 Health and Social Care Act at the onset of devolution. This likely contributed to the influence that groups could have on proceedings. These are unintended outcomes that have been rarely discussed in public health research. In this regard, the findings are further examples of reorganization with the promise of more emphasis on prevention, which has largely left ‘true’ prevention practices unchanged in the initial years, and perhaps even more marginalized in some cases. This can be explained through interweaving processes that included medical prestige, the ideological and political dominance of individualism, and an overwhelming need for treatment services, all in the face of financial constraint. This study in GM shows that such complexities can mean that many, even those with considerable personal commitment for change, are likely to retreat towards a continuation of the norm, with little encouragement, in policy terms, to do otherwise. As acknowledged by the Chief Officer of the GMH&SCP: ‘For all the benefits of formality, decision-making in the devolved system can be slow at times. There is still an understandable tendency to hide behind a sense of fiduciary duty to one’s own organization rather than what is genuinely in the public interest for GM as a whole’^53^ (p. 4). This all resulted in a clear double-bind whereby devolution ‘promised’ an alternative way of doing things, yet participants described doubling down on ‘old’ practices, especially when considering the marginalization and stigmatization of non-clinical practitioners.

### Study limitations

There is much continuity alongside change in public health policy (and wider devolution) fields, as associated networks continue to expand. Interviews for this study were conducted within a two-year period, during the first phase of devolution in GM, however, it may have been advantageous to conduct data collection over a longer period to track new developments. Furthermore, there may be aspects of our findings that are somewhat unique to networks in GM. However, we argue that our analysis of key processes related to the implementation of health devolution in GM are very likely to have relevance for other contexts. In particular, theoretical insights from this study, such as the increased complexity of devolution, and medical prestige, continue to have currency given the expanding number of regions leading the implementation of newly devolved powers, likewise regions seeking to elevate their work on prevention.

## Data Availability

Further raw data cannot be shared for ethical/privacy reasons. We have agreed to only share data that pertains to academic need/publication.

## Notes

**Chris White**, Senior Lecturer Health, Mental Health, and Wellbeing

**Daniel Bloyce**, Head of Division Public Health, Sport, and Wellbeing

**Miranda Thurston**, Professor of Public Health

**Paul Bissell**, Pro Vice-Chancellor Research and Innovation

## References

[1] Baum FE, Legge DG, Freeman T et al. The potential for multi-disciplinary primary health care services to take action on the social determinants of health: actions and constraints. BMC Public Health 2013;13:1–3. 10.1186/1471-2458-13-460 23663304 PMC3660265

[2] Blue S, Shove E, Carmona C et al. Theories of practice and public health: understanding (un) healthy practices. Crit Public Health 2016;26:36–50. 10.1080/09581596.2014.980396

[3] McMahon NE . Framing action to reduce health inequalities: what is argued for through use of the ‘upstream–downstream’ metaphor? J Public Health 2022;44:671–8. 10.1093/pubmed/fdab157 PMC9424054 34056659

[4] Szreter S, Woolcock M. Health by association? Social capital, social theory, and the political economy of public health. Int J Epidemiol 2004;33:650–67. 10.1093/ije/dyh013 15282219

[5] Ige-Elegbede J, Pilkington P, Orme J et al. Designing healthier neighbourhoods: a systematic review of the impact of the neighbourhood design on health and wellbeing. Cities Health 2022;6:1004–19. 10.1080/23748834.2020.1799173 36618774 PMC9810039

[6] Dahlgren G, Whitehead M. The Dahlgren-Whitehead model of health determinants: 30 years on and still chasing rainbows. Public Health 2021;199:20–4. 10.1016/j.puhe.2021.08.009 34534885

[7] Dean HD, Williams KM, Fenton KA. From theory to action: applying social determinants of health to public health practice. Public Health Rep 2013;128:1–4. 10.1177/00333549131286S301 PMC3945442 24179272

[8] Greer SL, Wismar M, Kosinska M. Towards intersectoral governance: lessons learned from health system governance. Public Health Panor 2015;1:128–32.

[9] Marmot M . *Fair Society, Healthy Lives: The Marmot Review*. London: Institute of Health Equity, 2010.

[10] Marmot M . Health equity in England: the Marmot review 10 years on. BMJ 2020;368:m693. 10.1136/bmj.m693 32094110

[11] Asthana S, Gibson A, Halliday J. The medicalisation of health inequalities and the English NHS: the role of resource allocation. Health Place 2013;8:167–83. 10.1017/S1744133112000126 22947257

[12] Goldberg DS . The perils of medicalization for population health and health equity. Milbank Q 2021;99:286–302. 10.1111/1468-0009.12619 PMC10126964 37096631

[13] Bevan G, Hood C. What's measured is what matters: targets and gaming in the English public health care system. Public Adm 2006;84:517–38. 10.1111/j.1467-9299.2006.00600.x

[14] Cairney J, McGannon KR, Atkinson M. Exercise is medicine: critical considerations in the qualitative research landscape. Qual Res Sport Exerc Health 2018;10:391–9. 10.1080/2159676X.2018.1476010

[15] Marshall T, Lowther R, Saunders P et al. Can targeting health inequalities really work? Lancet Public Health 2024;9:e88–9.38134944

[16] Edwards N, Black S. Targets: unintended and unanticipated effects. BMJ Qual Saf 2023;32:697–9. 10.1136/bmjqs-2023-016247 37669875

[17] Coalter F . *A Wider Social Role for Sport: Who’s Keeping the Score?* London: Routledge, 2007, 10.4324/9780203014615.

[18] Zola IK . Medicine as an institution of social control. Sociol Rev 1972;20:487–504. 10.1111/j.1467-954X.1972.tb00220.x 4645802

[19] Busfield J . The concept of medicalisation reassessed. Sociol Health Illn 2017;39:759–74. 10.1111/1467-9566.12538 28052343

[20] Lusardi R . Current trends in medicalisation: universalising ADHD diagnosis and treatments. Sociol Compass 2019;13:e12697. 10.1111/soc4.12697

[21] Crawford R . Healthism and the medicalization of everyday life. Int J Health Serv 1980;10:365–88. 10.2190/3H2H-3XJN-3KAY-G9NY 7419309

[22] Boni Z . Slim choices: young people’s experiences of individual responsibility for childhood obesity. Crit Public Health 2022;32:322–32. 10.1080/09581596.2020.1851655

[23] Mead R, Thurston M, Bloyce D. From public issues to personal troubles: individualising social inequalities in health within local public health partnerships. Crit Public Health 2022;32:168–80. 10.1080/09581596.2020.1763916

[24] Scott-Samuel A, Bambra C, Collins C et al. The impact of Thatcherism on health and well-being in Britain. Int J Health Serv 2014;44:53–71. 10.2190/HS.44.1.d 24684084

[25] Jones L, Hameiri S. COVID-19 and the failure of the neoliberal regulatory state. Rev Int Polit Econ 2022;29:1027–52. 10.1080/09692290.2021.1892798

[26] Mudge SL . What is neo-liberalism? Socio-Econ Rev 2008;6:703–31. 10.1093/ser/mwn016

[27] Schrecker T . Neoliberalism and health: the linkages and the dangers. Sociol Compass 2016;10:952–71. 10.1111/soc4.12408

[28] Brown S, Shoveller J, Chabot C et al. Risk, resistance and the neoliberal agenda: young people, health and well-being in the UK. Canada and Australia Health Risk Soc 2013;15:333–46. 10.1080/13698575.2013.796346

[29] Peacock M, Bissell P, Owen J. Dependency denied: health inequalities in the neo-liberal era. Soc Sci Med 2014;118:173–80. 10.1016/j.socscimed.2014.08.006 25137636

[30] Scott-Samuel A, Smith KE. Fantasy paradigms of health inequalities: utopian thinking? Soc Theory Health 2015;13:418–36. 10.1057/sth.2015.12

[31] Popay J, Whitehead M, Hunter DJ. Injustice is killing people on a large scale—but what is to be done about it? J Public Health 2010;32:148–9. 10.1093/pubmed/fdq029 20423920

[32] Powell K, Thurston M, Bloyce D. Theorising lifestyle drift in health promotion: explaining community and voluntary sector engagement practices in disadvantaged areas. Crit Public Health 2017;27:554–65. 10.1080/09581596.2017.1356909

[33] Greater Manchester Health & Social Care Partnership . *Taking Charge: The Next Steps for Health and Social Care in Greater Manchester*. Manchester: GMH&SCP, 2015.

[34] Ministry of Housing . *Communities & Local Government*. English Devolution White Paper. London: MHCLG, 2025.

[35] Katikireddi SV, Smith KE, Stuckler D et al. Devolution of power, revolution in public health? J Public Health 2017;39:241–7. 10.1093/pubmed/fdw031 PMC5892858 27165668

[36] Walshe K, Coleman A, McDonald R et al. *Health and Social Care Devolution: The Greater Manchester Experiment*. Manchester: University of Manchester, 2018. 10.1136/bmj.i1495 27006416

[37] Kelly MP, Atkins L, Littleford C et al. Reforming public health in England: the 2012 health and social care act. J Public Health 2017;39:421–5.

[38] Nuffield Trust . *The 2012 Health and Social Care Act: A Review*. London: Nuffield Trust, 2015.

[39] Gadsby E, Peckham S, Coleman A et al. *Commissioning for Better Outcomes: A Route Map*. London: Local Government Association, 2017.

[40] Gorsky M, Lock K, Hogarth S. *Public Health Returning Home*. London: Shared Intelligence, 2014.

[41] Insall P . Active travel, public health and the health and social care act. J Transp Health 2014;1:15–6.

[42] Lorne C, McDonald R, Walshe K et al. Regional assemblage and the spatial reorganisation of health and care: the case of devolution in greater Manchester, England. Sociol Health Illn 2019;41:1236–50. 10.1111/1467-9566.12867 30761548 PMC6833925

[43] Office for National Statistics . *UK Population Estimates by Region and Devolved Administration: Mid-2017*. London: ONS, 2019.

[44] Vize R, freelance journalist. The greater Manchester experiment. BMJ 2016;352:i1613. 10.1136/bmj.i1611 27004912

[45] Phillipson C . *Developing a Strategy for Age-Friendly Greater Manchester*. Manchester: University of Manchester, 2017.

[46] Musselwhite C . Prioritising transport barriers and enablers to mobility in later life: a case study from greater Manchester. J Transp Health 2021;22:101085. 10.1016/j.jth.2021.101085

[47] White C. Understanding active travel as a public health issue in Greater Manchester: A figurational sociology study [PhD thesis]. Chester, University of Chester; 2023.

[48] Roulston K . *Reflective Interviewing: A Guide to Theory and Practice*, 2nd edn. London: Sage, 2010, 10.4135/9781446288009.

[49] Braun V, Clarke V. Using thematic analysis in psychology. Qual Res Psychol 2006;3:77–101. 10.1191/1478088706qp063oa

[50] Braun V, Clarke V. Reflecting on reflexive thematic analysis. Qual Res Sport Exerc Health 2019;11:589–97. 10.1080/2159676X.2019.1628806

[51] Levitt HM, Bamberg M, Creswell JW et al. Journal article reporting standards for qualitative research in psychology. Am Psychol 2018;73:26–46. 10.1037/amp0000151 29345485

[52] Etherington D, Jones M. Re-stating the post-political: Depoliticization, social inequalities, and city-region growth. Environment and Planning A: Economy and Space. 2018;50:51–72.

[53] Rouse J . *Devolution, Population Health and the Greater Manchester Experiment [Public Lecture]*. Manchester: University of Manchester, 2020.

[54] Le Gouais A . A qualitative study exploring how local politicians influence healthy local planning policies. Cities Health 2025. [Epub ahead of print];10:122–31. 10.1080/23748834.2025.2493409

[55] Le Gouais A, Panter J, Cope A et al. Decision-making for active living infrastructure in new communities. J Public Health 2020;42:e249–58. 10.1093/pubmed/fdz105 PMC7435215 31565741

[56] Dopson S, Locock L. The commissioning process in the NHS: the theory and application. Public Manag Rev 2002;4:209–29. 10.1080/14616670210130552

[57] Lefebvre C, Chahine S, Al-Azzawi M et al. Determinants of medical specialty competitiveness. Postgrad Med J 2020;96:511–4. 10.1136/postgradmedj-2019-137160 31780597

